# Triglyceride to high-density lipoprotein cholesterol ratio is associated with regression to normoglycemia from prediabetes in adults: a 5-year cohort study in China

**DOI:** 10.1186/s12967-023-04752-w

**Published:** 2023-11-30

**Authors:** Zhiqiang Huang, Yong Han, Haofei Hu, Changchun Cao, Dehong Liu, Zhibin Wang

**Affiliations:** 1 Department of Emergency, Futian District, Shenzhen Second People’s Hospital, No. 3002 Sungang Road, Shenzhen, 518000 Guangdong Province China; 2 Department of Nephrology, Guangdong Province, Shenzhen Second People’s Hospital, Shenzhen, 518000 China; 3 Department of Rehabilitation, Shenzhen Dapeng New District Nan’ao People’s Hospital, Shenzhen, 518000 Guangdong Province China

**Keywords:** Prediabetes, Triglycerides-to-high-density lipoprotein cholesterol ratio, Regression to normoglycemia, Non-linear, Competitive risk model

## Abstract

**Objective:**

The current body of evidence on the association between the ratio of triglycerides to high-density lipoprotein cholesterol (TG/HDL-c) and the reversal of prediabetes to normoglycemia remains limited. The aim of this study is to investigate the association between TG/HDL-c and the reversion to normoglycemia in patients with prediabetes.

**Methods:**

This retrospective cohort study included 15,107 individuals with prediabetes from 32 Chinese districts and 11 cities who completed health checks from 2010 to 2016. The Cox proportional-hazards regression model examined baseline TG/HDL-c and reversion to normoglycemia from prediabetes. Cox proportional hazards regression with cubic spline functions and smooth curve fitting determined the non-linear connection between TG/HDL-c and reversion to normoglycemia. We also ran sensitivity and subgroup analysis. By characterizing progression to diabetes as a competing risk for the reversal of prediabetes to normoglycemic event, a multivariate Cox proportional hazards regression model with competing risks was created.

**Results:**

Upon adjusting for covariates, the findings indicate a negative association between TG/HDL-c and the likelihood of returning to normoglycemia (HR = 0.869, 95%CI:0.842–0.897). Additionally, a non-linear relationship between TG/HDL-c and the probability of reversion to normoglycemia was observed, with an inflection point of 1.675. The HR on the left side of the inflection point was 0.748 (95%CI:0.699, 0.801). The robustness of our results was confirmed through competing risks multivariate Cox's regression and a series of sensitivity analyses.

**Conclusion:**

The present study reveals a negative and non-linear correlation between TG/HDL-c and the reversion to normoglycemia among Chinese individuals with prediabetes. The findings of this study are anticipated to serve as a valuable resource for clinicians in managing dyslipidemia in prediabetic patients. Interventions aimed at reducing the TG/HDL-c ratio through the reduction of TG or elevation of HDL-c levels may substantially enhance the likelihood of achieving normoglycemia in individuals with prediabetes.

**Supplementary Information:**

The online version contains supplementary material available at 10.1186/s12967-023-04752-w.

## Introduction

The prevalence of diabetes presents a noteworthy public health issue due to its substantial morbidity, mortality, and increasing expenses [1]. Prediabetes serves as an intermediary state between typical blood sugar levels and type 2 diabetes (T2DM), frequently marked by impaired fasting glucose (IFG) or impaired glucose tolerance (IGT) [2]. The International Diabetes Federation (IDF) reported in 2017 that prediabetes affects 374 million individuals worldwide, with a projected increase to 548 million by 2045, representing 8.4% of adults [3]. In the United States, approximately 86 million people, accounting for about 37% of the population, have prediabetes [4]. The incidence of prediabetes among Chinese adults stands at around 35.7% [5]. As per the expert panel of the American Diabetes Association (ADA), individuals diagnosed with prediabetes are at a heightened risk of developing T2DM, with an estimated annual progression rate of 5–10% and a potential development rate of up to 70% [6, 7]. However, it is noteworthy that not all individuals with prediabetes progress to diabetes; some remain in the prediabetic stage, and a significant percentage of prediabetic individuals, ranging from 20 to 50%, may even regress to normoglycemia [7–10]. A previous study has demonstrated that even a transient return to normal blood sugar levels in patients with prediabetes is associated with a substantial reduction in the risk of developing diabetes [11]. Therefore, the therapeutic value of reversing prediabetes and achieving normoglycemia cannot be overstated. Treatment strategies for prediabetes should prioritize the normalization of blood sugar levels. Identifying contributing factors to the reversion from prediabetes to normoglycemia is particularly important in identifying preventive pathways to impede the progression from prediabetes to diabetes and establishing actionable goals for public health interventions.

Unfortunately, the current focus of clinical attention seems to be primarily on disease development, with only a limited number of studies investigating the frequency of regression to normoglycemia in patients with prediabetes and the influencing risk factors. Preliminary data from earlier epidemiological research suggest that age, insulin secretion, baseline fasting glucose, β-cell function, obesity, and fasting triglycerides may be associated with the return to normoglycemia [4, 12–15]. Previous studies have confirmed a significant relationship between dyslipidemia and dysglycemia [16, 17]. Dyslipidemia is typified by the presence of diminished levels of high-density lipoprotein cholesterol (HDL-c) or heightened concentrations of low-density lipoprotein cholesterol (LDL-c) and triglycerides (TG). Recently, the ratio of triglycerides to high-density lipoprotein cholesterol (TG/HDL-c) has been identified as a measure of harmful lipid imbalance, linked to various health risks. These include cardiovascular events, insulin resistance(IR), high blood pressure, fatty liver, and diabetes [18–20]. The relationship between the TG/HDL-c ratio and the progression from prediabetes to diabetes has been explored in a single study [21]. In addition, Furthermore, multiple studies have indicated a positive correlation between TG/HDL-c and diabetes risk in the general population [22–26]. Given that TG/HDL-c is a significant risk factor for diabetes, we put forth the conjecture that there may be a negative association between TG/HDL-c and the probability of returning to normoglycemia from prediabetes. Indeed, the research exploring the link between the TG/HDL-c ratio and the transition from prediabetes back to normal blood sugar levels is sparse, with only a handful of studies tackling this issue. In this retrospective cohort study, our objective is to delve into the relationship between the TG/HDL-c ratio and the probability of reverting to normoglycemia from prediabetes using the available published data.

## Methods

### Study design

A retrospective cohort study design was utilized in this investigation. The data were obtained from an existing retrospective cohort study database established by Chinese researchers (Chen et al.) [27]. The independent variable was the TG/HDL-c ratio, which was assessed at baseline. The dependent variable was the regression from prediabetes to normoglycemia during the follow-up period.

### Data source

The data utilized in this study were sourced from DATADRYAD (www.datadryad.org) and generously provided by Ying Chen et al. [27]. The dataset was extracted from a published article entitled "Association of body mass index and age with incident diabetes in Chinese adults: a population-based cohort study" (http://dx.doi.org/10.1136/bmjopen-2018-021768) [27]. This article was an open-access publication licensed under the Creative Commons Attribution Non-Commercial (CC BY-NC 4.0) license, which permits the sharing, remixing, modification, and creation of derivative works of this material for non-commercial purposes, with proper credit given to the author and source [27].

### Study population

The information was obtained from a computerized database created by China's Rich Healthcare Group Medical Group, as indicated by the initial researchers [27]. This comprehensive database contained medical records of participants who underwent health screenings between 2010 and 2016, encompassing 32 regions and 11 cities across China. The original study received prior approval from the Rich Healthcare Group review committee, and the data were retrospectively retrieved. For this retrospective study, there was no requirement for informed consent or approval from the institutional ethics committee [27]. As a result, the secondary analysis did not necessitate ethical approval.

The original study enrolled a total of 685,277 individuals aged 20 and above who underwent at least two health screenings, with 473,444 individuals excluded based on the exclusion criteria. The original study implemented the following exclusion criteria: (I) participants with less than two years between visits; (II) individuals with an extremely high or low BMI (less than 15 kg/m^2^ or greater than 55 kg/m^2^); (III) participants lacking baseline information on height, sex, weight, and fasting plasma glucose (FPG) values; (IV) individuals with diabetes at baseline; and (V) participants with unknown diabetes status during follow-up. Ultimately, the analysis of the original study included 211,833 individuals.

**Fig. 1:**
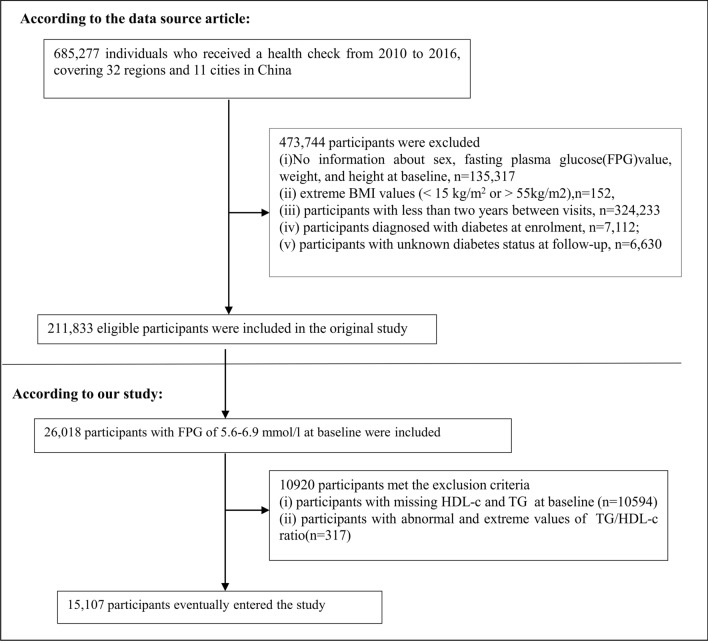
Flowchart of study participants

In the present study, we expanded the participant pool by including an additional 26,018 individuals with a baseline FPG level ranging from 5.6 to 6.9 mmol/L, which aligns with the criteria for prediabetes according to the 2021 guidelines of the ADA. Subsequently, we excluded participants with missing data on HDL-c and TG at baseline (n = 10,594), as well as those with extreme and abnormal TG/HDL-c exceeding or falling below three standard deviations from the mean (n = 317). Finally, the total number of participants in the current study was 15,107. Fig. 1 depicted the procedure for participant selection.

## Variables

### Independent variable

The TG/HDL-c ratio was identified as a continuous variable. The TG/HDL-c ratio is computed as TG divided by HDL-c.

### Outcome variable

Our primary outcome variable of interest was the occurrence of regression to normoglycemia. The determination of normoglycemia recovery was based on two criteria: Participants did not self-report having diabetes at the follow-up assessment and had an FPG ≤ 5.6 mmol/L [10, 28].

### Covariates

The selection of variables for our study was based on the original study, studies pertaining to prediabetes or diabetes, and clinical expertise [12, 27, 29–31]. The covariates included both categorical and continuous variables. The categorical variables consisted of sex, family history of diabetes, smoking status, and alcohol consumption status. On the other hand, the continuous variables encompassed body weight, serum creatinine (Scr), body mass index (BMI), age, height, aspartate aminotransferase (AST), TG, systolic blood pressure (SBP), HDL-c, alanine aminotransferase (ALT), blood urea nitrogen (BUN), diastolic blood pressure (DBP), total cholesterol (TC), and as well as LDL-c.

### Data collection

In the initial study, questionnaires were used to gather basic information on alcohol intake, smoking habits, and diabetes family history. Blood pressure was noted using a standard mercury sphygmomanometer. After fasting for at least 10 h, fasting venous blood samples were drawn from participants. A Beckman 5800 autoanalyzer was then used to assess levels of TG, AST, FPG, HDL-c, BUN, TC, Scr, LDL-c, and ALT [27].

### Treatment of missing data

There were 354 (2.34%), 5 (0.03%), 35 (0.23%), 26 (0.17%), 5 (0.03%), 113 (0.75%), 10,472 (69.32%), 8120 (53.75%), and 10,472 (69.32%) participants lacking data for BUN, DBP, ALT, LDL-c, SBP, Scr, smoking status, and AST in the current study, respectively. In this study, multiple imputations were performed to address missing data and lessen the uncertainty caused by missing variables [32, 33]. Family history of diabetes, BMI, TG, BUN, age, ALT, smoking status, Scr, sex, AST, DBP, SBP, TC, and drinking status was included in the inferential model (number of iterations 10; regression type linear).The missing randomization (MAR) assumption was used during the missing data analysis [33].

### Statistical analysis

Participants were stratified into four categories based on the quartile of TG/HDL-c ratio [34]. Continuous variables were expressed as mean (standard deviation SD) for Gaussian distribution variables and median (interquartile) for skewed distribution variables. Categorical variables were expressed as frequencies and percentages. The differences between groups were evaluated using the χ2 test for categorical variables, the one-way ANOVA test for normally distributed variables, and the Kruskal–Wallis H test for skewed variables.

### Multivariate cox proportional-hazards regression analysis

TG/HDL-c and regression to normoglycemia in individuals with prediabetes were examined using univariate and multivariate Cox proportional-hazards regression models. Three models were used: a crude model with no adjusted covariates, a minimally adjusted model (BMI, sex, and age), and Model II with full covariate adjustments (sex, BMI, age, drinking status, FPG, ALT, LDL-c, DBP, Scr, AST, BUN, family history of diabetes, smoking status, and SBP). Clinical experience, literature, and univariate analysis controlled confounding factors. Because of collinearity, TC was removed from the multivariate Cox proportional hazards regression equation (Additional file 1: Table S1).

Considering that individuals who develop diabetes during the follow-up period are improbable to undergo a reversion to normoglycemia from prediabetes, the observation of prediabetes regression events may be affected, or the probability of such events occurring may be influenced [30, 35, 36]. Consequently, a competing risk multivariate Cox proportional-hazards regression was conducted, following the methodology outlined by Fine and Gray [37, 38]. In this analysis, the progression to diabetes was regarded as the competing risk for reversing normoglycemia events.

Besides, the exploration of the non-linear association between TG/HDL-c and the rate of return to normoglycemia from prediabetes was facilitated through Cox proportional hazards regression with cubic spline functions and smooth curve fitting. The discovery of the inflection point in the event of a non-linear relationship was enabled by a recursive algorithm. A two-piecewise linear regression model was employed to determine the threshold effect. Lastly, a log-likelihood ratio test was conducted to determine the optimal model for describing their relationship.

### Subgroup analyses

Subgroup analyses were conducted on different subgroups (smoking status, sex, BMI, age, SBP, and drinking status) using stratified Cox proportional hazards regression models. Initially, continuous variables, including SBP, BMI, and age were transformed into categorical variables according to clinical cut-off factors (BMI: < 18.5, ≥ 18.5 to < 25, ≥ 25 kg/m^2^; SBP: 140, 140 mmHg; age: < 30 years, 30 to 40 years, 40 to 50 years, 50 to 60 years, 60 to 70 years, ≥ 70 years;)[39, 40]. Apart from the stratification factors themselves, each stratum was adjusted for SBP, sex, smoking status, AST, BMI, age, DBP, BUN, FPG, LDL-c, ALT, Scr, family history of diabetes, and drinking status.

### Sensitivity analyses

In order to ensure the validity and reliability of our research outcomes, a set of sensitivity analyses were performed. Initially, the TG/HDL-c ratio was transformed into a categorical variable based on quartiles, and the P for trend was computed to examine using TG/HDL-c as a continuous variable and to explore the possibility of nonlinearity. Prior research demonstrated a significant relationship between obesity, diabetes history, alcohol consumption and glucose metabolism [41–43]. Thus, among other sensitivity analyses, participants with a BMI > 28 kg/m^2^ were excluded from investigating the link between TG/HDL-c and the reversal of prediabetes to normoglycemia. Additionally, sensitivity analyses were performed by excluding participants with a family history of diabetes and current or past alcohol drinkers. Furthermore, a generalized additive model (GAM) was utilized to incorporate continuous covariates as curves into the equation to ensure the robustness of the results. Moreover, the possibility of unmeasured confounders between TG/HDL-c and the reversal of prediabetes to normoglycemia was explored by calculating the E-value [44, 45].

The STROBE statement was adhered to in documenting all findings. Empower Stats (X&Y Solutions, Inc., Boston, MA, http://www.empowerstats.com; version: 2.0) and the R statistical software packages (http://www.r-project.org, The R Foundation; version: 4.3.1) were utilized for all statistical analyses. A two-sided significance level of 0.05 was employed to ascertain statistical significance. The R code for the entire statistical analysis process is detailed in Additional file 2.

## Results

### Characteristics of patients with prediabetes

**Fig. 2:**
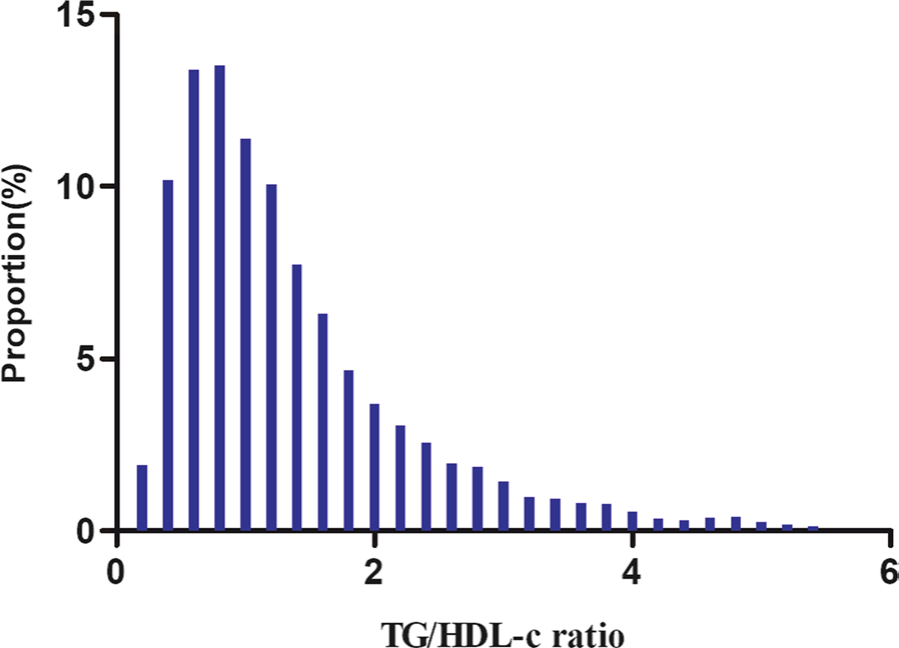
Distribution of TG/HDL-c ratio. It presented a skewed distribution, ranging from 0.04 to 5.45, with a mean of 1.35

Table 1 depicted the demographic and clinical characteristics of individuals diagnosed with prediabetes. The mean age of the participants was 50.95 ± 13.48 years, with 9745 (64.51%) of them being male. The median duration of follow-up was 2.95 years, during which 6,332 (41.91%) participants reverted to normoglycemia. The number of lost to follow-up in this study was 6,588, for a total of 43.61% lost to follow-up. The TG/HDL-c ratio displayed a skewed fractional distribution, with a median (interquartile range) of 1.09 (0.69–1.72) (Fig. 2). The participants were classified into subgroups based on the quartiles of TG/HDL-c (Q1: < 0.692, Q2:0.692–1.093, Q3:1.093–1.719, Q4: ≥ 1.719). Compared to participants in the Q1 group, participants in the Q4 group had significant increases in TG/HDL-c, age, TG, weight, LDL-c, ALT, SBP, BMI, height, DBP, TC, FPG, Scr, and AST, while there was an opposite trend in HDL-c. In addition, the Q1 group had a higher proportion of women and never drinkers.

**Table 1:** The Baseline characteristics of participants

TG/HDL ratio quartile	Q1(< 0.692)	Q2(0.692–1.093)	Q3(1.093–1.719)	Q4(≥ 1.719)	P-value
Participants	3764	3789	3777	3777	
Sex					< 0.001
Male	1784 (47.40%)	2368 (62.50%)	2672 (70.74%)	2921 (77.34%)	
Female	1980 (52.60%)	1421 (37.50%)	1105 (29.26%)	856 (22.66%)	
Weight(kg)	62.44 ± 10.62	68.17 ± 11.17	71.43 ± 11.58	74.43 ± 11.79	< 0.001
Height(cm)	164.63 ± 8.21	166.25 ± 8.46	167.21 ± 8.35	168.32 ± 8.05	< 0.001
Age(years)	48.68 ± 13.87	51.03 ± 13.96	52.11 ± 13.19	51.96 ± 12.60	< 0.001
DBP (mmHg)	75.25 ± 10.78	77.92 ± 11.11	79.64 ± 11.12	80.92 ± 10.90	< 0.001
FPG(mmol/L)	5.90 ± 0.29	5.93 ± 0.30	5.98 ± 0.33	6.01 ± 0.34	< 0.001
TG (mmol/L)	0.76 ± 0.21	1.22 ± 0.24	1.74 ± 0.36	2.99 ± 1.05	< 0.001
SBP (mmHg)	123.13 ± 17.59	127.36 ± 17.66	129.11 ± 17.37	130.12 ± 17.37	< 0.001
TC (mmol/L)	4.79 ± 0.89	4.98 ± 0.92	5.11 ± 0.93	5.24 ± 0.95	< 0.001
AST(U/L)	23.04 ± 10.76	25.36 ± 11.65	27.07 ± 11.29	29.16 ± 12.28	< 0.001
LDL-c(mmol/L)	2.78 ± 0.67	2.96 ± 0.68	3.03 ± 0.70	3.01 ± 0.76	< 0.001
Scr (μmol/L)	68.80 ± 15.45	72.71 ± 16.44	74.66 ± 15.80	75.65 ± 16.13	< 0.001
ALT(U/L)	16.30 (12.17–23.00)	20.70 (15.00–29.20)	24.00 (17.10–35.00)	28.50 (20.00–41.60)	< 0.001
HDL-c(mmol/L)	1.56 ± 0.32	1.39 ± 0.23	1.28 ± 0.23	1.13 ± 0.24	< 0.001
BUN (mmol/L)	4.98 ± 1.25	5.03 ± 1.26	5.03 ± 1.23	4.98 ± 1.21	0.095
TG/HDL ratio	0.49 ± 0.13	0.88 ± 0.12	1.37 ± 0.18	2.67 ± 0.85	< 0.001
Smoking status					0.037
Current smoker	878 (23.33%)	938 (24.76%)	962 (25.47%)	1003 (26.56%)	
Ever smoker	174 (4.62%)	186 (4.91%)	197 (5.22%)	186 (4.92%)	
Never smoker	2712 (72.05%)	2665 (70.34%)	2618 (69.31%)	2588 (68.52%)	
Drinking status					< 0.001
Current drinker	181 (4.81%)	226 (5.96%)	146 (3.87%)	200 (5.30%)	
Ever drinker	688 (18.28%)	747 (19.71%)	781 (20.68%)	728 (19.27%)	
Never drinker	2895 (76.91%)	2816 (74.32%)	2850 (75.46%)	2849 (75.43%)	
Family history of diabetes					0.796
No	3674 (97.61%)	3686 (97.28%)	3682 (97.48%)	3676 (97.33%)	
Yes	90 (2.39%)	103 (2.72%)	95 (2.52%)	101 (2.67%)	

Continuous variables are summarized as the mean (standard deviation) or median (interquartile range), whereas categorical variables are presented as percentages (%)

DBP, diastolic blood pressure; *HDL-c* high-density lipoprotein cholesterol, *Scr* serum creatinine, *TG* triglyceride, *SBP* systolic blood pressure, *TC* total cholesterol, *BMI* body mass index, *ALT* alanine aminotransferase, *LDL-c* low-density lipoprotein cholesterol, *BUN* blood urea nitrogen, *TG/HDL-c* *ratio* triglyceride-to-high-density lipoprotein cholesterol ratio, *AST* aspartate aminotransferase

In the provided Additional file 1: Table S2, baseline characteristics of patients with prediabetes, including their regression and progression status, are presented. It was observed that patients who progressed to diabetes exhibited higher values of age, weight, BMI, DBP, SBP, TG, LDL-c, TC, AST, ALT, Scr, and BUN, and a decreased HDL-c level compared to persistently prediabetic patients. Conversely, lower measurements of age, weight, BMI, DBP, SBP, TG, LDL-c, TC, AST, ALT, Scr, and BUN were found in patients who reverted to normoglycemia, coupled with an elevated HDL-c level, in comparison to persistently prediabetic individuals.

### Reversal rate from prediabetes to normoglycemia

**Fig. 3:**
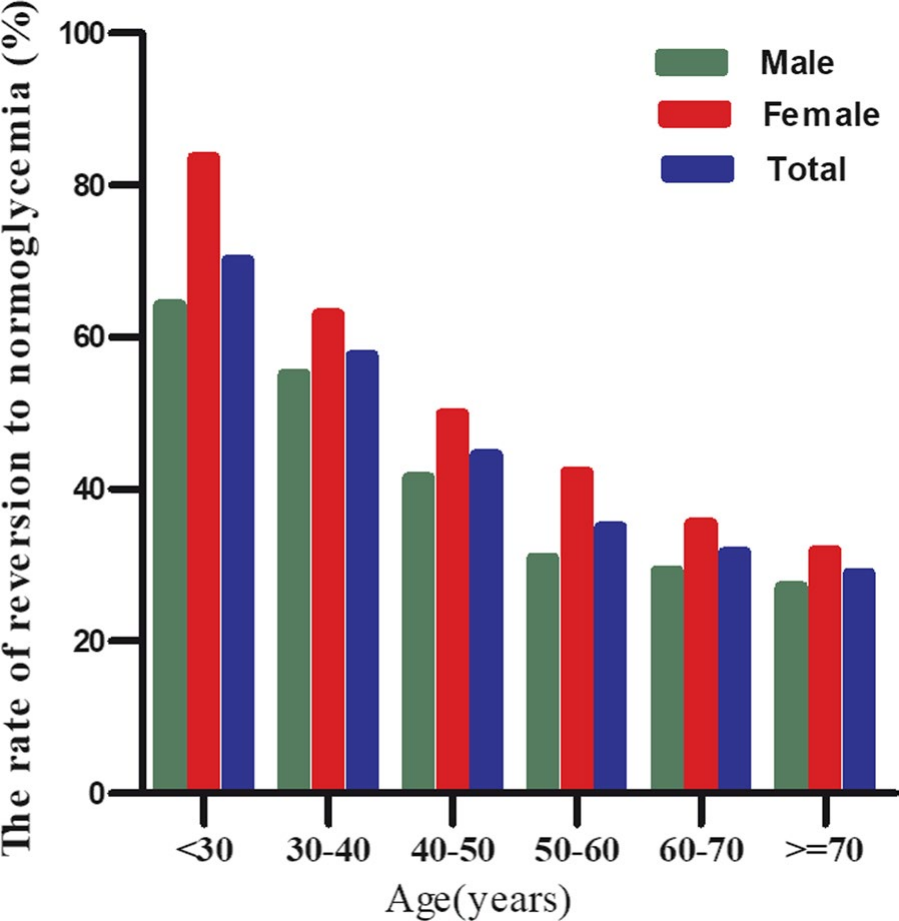
The rate of reversion to normoglycemia in prediabetic patients of age stratification by 10 intervals. The rate of regression to normoglycemia among participants with prediabetes was higher in women than in men, regardless of their age group. It was also found that the reversal rate decreased with age in both men and women

During a median follow-up time of 2.95 years, a total of 6332 participants experienced a reversal to normoglycemia. The overall rate of regression to normoglycemia was 142.21 per 1,000 person-years. Among patients with prediabetes, the quartiles of TG/HDL-c ratio were associated with rates of regression to normoglycemia as follows: Q1: 179.85, Q2: 155.35, Q3: 122.95, and Q4: 113.15 per 1,000 person-years. A cumulative regression rate of 41.91% was observed within 2.95 years, with respective quartile regressions showing Q1:51.54%, Q2:45.05%, Q3:36.33%, and Q4:34.76%. The fourth quartile presented lower reversion rates significantly compared to the first quartile (P < 0.001 for trend) (Additional file 1: Table S3 and Figure S1). In the analysis stratified by age intervals of 10 years, it was observed that the probability of regression to normoglycemia among participants with prediabetes was higher in women than men across all age groups. Additionally, it was noted that the rates of reversal decreased with advancing age in both women and men. (Fig. 3).

### Univariate Cox proportional hazards regression was used to examine the factors influencing the reversion to normoglycemia

The results of the univariate analyses indicate that there were no significant associations between the reversal rate to normoglycemia from prediabetes and LDL-c (HR = 0.990, 95% CI 0.956–1.025) or TC (HR = 0.984, 95% CI 0.958–1.011). However, a negative association was observed between the reversal rate to normoglycemia and age (HR = 0.980, 95% CI 0.978–0.982), BMI (OR = 0.934, 95% CI 0.927–0.942), DBP (HR = 0.985, 95% CI 0.982–0.987), SBP (HR = 0.988, 95% CI 0.987–0.990), FPG (HR = 0.224, 95% CI 0.202–0.248), ALT (HR = 0.993, 95% CI 0.992–0.995), AST (HR = 0.988, 95% CI 0.985–0.990), TG (HR = 0.819, 95% CI 0.796–0.843), BUN (HR = 0.958, 95% CI 0.939–0.978), and Scr (HR = 0.995, 95% CI 0.994–0.997) (all p < 0.05). Interestingly, HDL-c showed a positive relationship with the reversal rate to normoglycemia from prediabetes (HR = 2.015, 95% CI 1.887–2.153) (Table 2).

**Table 2:** Univariate Cox proportional hazards regression was used to examine the factors influencing the reversion to normoglycemia

	Statistics	HR (95% CI)	P-value
Age(years)	50.945 ± 13.482	0.980 (0.978, 0.982)	< 0.001
Sex			
Male	9745 (64.507%)	Ref	
Female	5362 (35.493%)	1.270 (1.208, 1.336)	< 0.001
BMI (kg/m^2^)	24.801 ± 3.315	0.934 (0.927, 0.942)	< 0.001
DBP (mmHg)	78.435 ± 11.178	0.985 (0.982, 0.987)	< 0.001
SBP (mmHg)	127.434 ± 17.695	0.988 (0.987, 0.990)	< 0.001
FPG (mmol/L)	5.952 ± 0.319	0.224 (0.202, 0.248)	< 0.001
TC (mmol/L)	5.029 ± 0.936	0.984 (0.958, 1.011)	0.248
HDL-c(mmol/L)	1.341 ± 0.300	2.015 (1.887, 2.153)	< 0.001
TG (mmol/L)	1.430 (0.990–2.100)	0.819 (0.796, 0.843)	< 0.001
TG/HDL ratio	1.093 (0.692–1.718)	0.770 (0.747, 0.794)	< 0.001
LDL-c(mmol/L)	2.944 ± 0.712	0.990 (0.956, 1.025)	0.572
ALT(U/L)	22.000 (15.400–32.500)	0.993 (0.992, 0.995)	< 0.001
AST(U/L)	24.206 (19.568–30.200)	0.988 (0.985, 0.990)	< 0.001
BUN (mmol/L)	5.004 ± 1.240	0.958 (0.939, 0.978)	< 0.001
Scr (μmol/L)	72.958 ± 16.170	0.995 (0.994, 0.997)	< 0.001
Drinking status			
Current drinker	753 (4.984%)	Ref	
Ever drinker	2944 (19.488%)	1.045 (0.920, 1.188)	0.496
Never	11410 (75.528%)	1.097 (0.974, 1.235)	0.126
Family history of diabetes			
No	14718 (97.425%)	Ref	
Yes	389 (2.575%)	0.875 (0.751, 1.021)	0.090
Smoking status			
Current smoker	3781 (25.028%)	Ref	
Ever smoker	743 (4.918%)	0.967 (0.855, 1.094)	0.596
Never	10583 (70.054%)	1.014 (0.957, 1.074)	0.636

Continuous variables are summarized as the mean (standard deviation) or median (interquartile range), whereas categorical variables are presented as percentages (%)

**Fig. 4:**
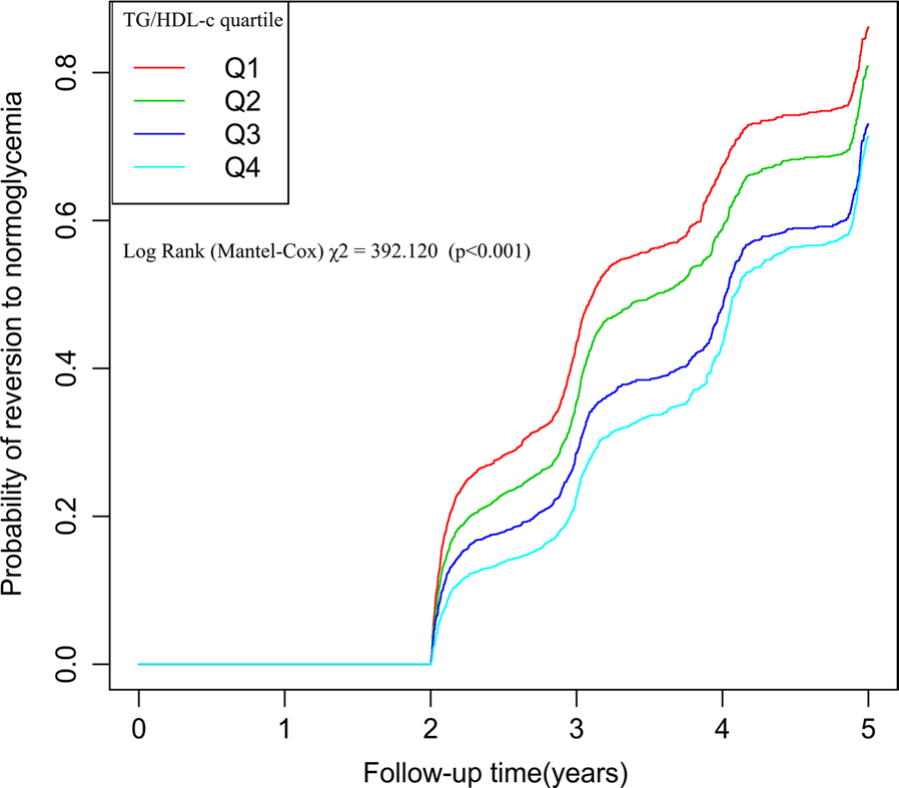
Kaplan–Meier curves for the probability of reversion to normoglycemia from prediabetes. The probability of reversion to normoglycemia decreased progressively with rising TG/HDL-c, meaning that Patients with the highest TG/HDL-c had the lowest probability of reverting from prediabetes to normoglycemia

Figure 4 revealed TG/HDL-c quartile-stratified Kaplan–Meier curves for the probability of reversal from prediabetes to normoglycemia. The regression probability from prediabetes to normoglycemia significantly differed among TG/HDL-c quartiles (log-rank test, P < 0.001). The reversal rate to normoglycemia decreased progressively as TG/HDL-c increased, implying that patients with the highest TG/HDL-c had the lowest probability of returning from prediabetes to normoglycemia.

### The association between the TG/HDL-c ratio and regression from prediabetes to normoglycemia was analyzed by multivariate cox proportional-hazards regression models

The crude model revealed that a 1-unit increase in the TG/HDL-c ratio was significantly associated with a 23% decrease in the likelihood of returning to normoglycemia (HR = 0.770, 95% CI 0.747–0.794). Upon adjusting for demographic variables in the minimally adjusted model, each 1-unit increase in the TG/HDL-c ratio was found to be linked to a 15.2% decrease in the probability of returning to normoglycemia (HR = 0.848, 95% CI 0.821–0.875). In the fully adjusted model, the HR between them was 0.869 (95% CI 0.842–0.897) (Table 3).

**Table 3:** Relationship between TG/HDL-c ratio and reversion to normoglycemia in patients with prediabetes in different models

Exposure	Crude (HR,95%CI)	Model I(HR,95%CI)	Model II(HR,95%CI)	Model III(HR,95%CI)
TG/HDL-c ratio	0.770 (0.747, 0.794) < 0.001	0.848 (0.821, 0.875) < 0.001	0.869 (0.842, 0.897) < 0.001	0.874 (0.846, 0.903) < 0.001
TG/HDL ratio quartiles				
Q1	Ref	Ref	Ref	Ref
Q2	0.807 (0.756, 0.861) < 0.001	0.919 (0.859, 0.983) 0.013	0.925 (0.864, 0.990) 0.02377	0.918 (0.857, 0.983) 0.01392
Q3	0.616 (0.575, 0.660) < 0.001	0.752 (0.699, 0.810) < 0.001	0.775 (0.719, 0.835) < 0.001	0.772 (0.715, 0.832) < 0.001
Q4	0.528 (0.492, 0.566) < 0.001	0.667 (0.617, 0.720) < 0.001	0.706 (0.653, 0.764) < 0.001	0.713 (0.658, 0.772) < 0.001
P for trend	< 0.001	< 0.001	< 0.001	< 0.001

Crude model: unadjusted for any variable

Model I: Adjusted variables included age, BMI, sex

Model II: Adjusted variables included age, SBP, BMI, sex, LDL-c, AST, DBP, FPG, ALT, Scr, family history of diabetes, BUN, smoking status, and drinking status

Model III: Adjusted variables included age(smooth), sex, BMI (smooth), ALT (smooth), FPG (smooth), BUN (smooth), SBP (smooth), LDL-c (smooth), DBP(smooth), AST(smooth), smoking status, Scr(smooth), drinking status, family history of diabetes

*HR*, Hazard ratios, *CI* confidence, *Ref* reference

Furthermore, we performed a categorical transformation of the TG/HDL-c ratio and included it in the multivariate-adjusted model. Comparing participants in the first quartile of the TG/HDL-c ratio as the reference group, the HR for participants in the second quartile was 0.925 (95% CI 0.864–0.990), indicating a 7.5% lower probability of returning to normoglycemia. For participants in the third quartile, the HR was 0.775 (95% CI 0.719–0.835), indicating a 22.5% lower probability. Finally, participants in the fourth quartile had an HR of 0.706 (95% CI 0.653–0.764). This indicated that participants in the fourth quartile (Q4) of the TG/HDL-c ratio had a 29.4% lower probability of returning to normoglycemia compared to those in the first quartile (Q1) (Table 3 Model II).

### The outcomes of a multivariate Cox proportional-hazards regression with competing risks

The results of the competing risks multivariate Cox proportional-hazards regression analysis, which investigated the progression of prediabetes to incident diabetes as a competing event for reversion to normoglycemia, are presented in Table 4. In the crude model, the analysis showed a negative association between TG/HDL-c and the probability of regression to normoglycemia, with a subdistribution hazard ratio (SHR) of 0.77 (95% CI 0.75–0.79). After adjusting for BMI, age, and sex (model I), the SHR for the TG/HDL-c ratio and the probability of returning to normoglycemia was 0.85 (95% CI 0.82–0.87). Importantly, even after further adjustments for additional variables in the fully adjusted model (model II), including BMI, ALT, DBP, sex, FPG, age, LDL-c, SBP, drinking status, AST, Scr, smoking status, BUN, and family history of diabetes, a negative association between them persisted (SHR = 0.87, 95% CI 0.84–0.90).

**Table 4:** Relationship between TG/HDL-c ratio and reversion to normoglycemia in patients with prediabetes in different models with competing risk of progression to diabetes

Exposure	Crude model (SHR,95%CI, P)	Model I(SHR,95%CI, P)	Model II (SHR,95%CI, P)
TG/HDL-c ratio	0.77 (0.75, 0.79) < 0.001	0.85 (0.82, 0.87) < 0.001	0.87 (0.84, 0.90) < 0.001
TG/HDL ratio quartiles			
Q1	Ref	Ref	Ref
Q2	0.81 (0.76, 0.86) < 0.001	0.92 (0.86, 0.98) < 0.001	0.92 (0.86, 0.99) 0.024
Q3	0.62 (0.58, 0.66) < 0.001	0.75 (0.70, 0.81) < 0.001	0.77 (0.72, 0.83) < 0.001
Q4	0.53 (0.49, 0.57) < 0.001	0.67 (0.62, 0.72) < 0.001	0.71 (0.65, 0.76) < 0.001
P for trend	< 0.001	< 0.001	< 0.001

Crude model: we did not adjust other covariates

Model I: Adjusted variables included age, BMI, sex,

Model II: Adjusted variables included age, SBP, BMI, sex, LDL-c, AST, DBP, FPG, ALT, Scr, family history of diabetes, BUN, smoking status, and drinking status

*SHR* sub-distribution hazard ratios, *Ref* reference, *CI* confidence

Furthermore, when TG/HDL-c ratio was used as a categorical variable, the results of the multivariate adjustment model (fully adjusted model) showed that individuals in the second, third, and fourth quartiles of TG/HDL-c ratio exhibited a decreased likelihood of returning to normoglycemia by 8% (SHR = 0.92, 95% CI 0.86–0.99), 23% (SHR = 0.77, 95% CI 0.72–0.83), and 29% (SHR = 0.71, 95% CI 0.65–0.76), respectively, in comparison to those in the first quartile.

### Sensitivity analysis

Initially, GAM was utilized to incorporate continuous covariates as curves into the equation. The findings of Model III, as presented in Table 3, were consistent with the fully adjusted Model II. Notably, individuals in the fourth quartile of TG/HDL-c demonstrated a reduced likelihood of returning to normoglycemia compared to patients with prediabetes in the first quartile, with a decrease of 28.7% (HR = 0.713, 95% CI 0.658–0.772).

Besides, a sensitivity analysis was conducted on a subset of participants who had abstained from alcohol consumption (n = 11,410). Confounding variables, including smoking status, BMI, sex, FPG, AST, LDL-c, age, Scr, DBP, ALT, BUN, SBP, and family history of diabetes, were adjusted for. The findings indicated an inverse association between TG/HDL-c and regression from prediabetes to normoglycemia (HR = 0.870, 95% CI 0.839–0.902, p < 0.001). Furthermore, a sensitivity analysis was performed by excluding individuals with a family history of diabetes (n = 14,718). After adjusting for confounding variables, including smoking status, BMI, sex, FPG, LDL-c, AST, age, Scr, DBP, ALT, BUN, SBP, and drinking status, the negative association between them persisted (HR = 0.869, 95% CI 0.841–0.897, p < 0.001). Additionally, the analysis was restricted to patients with a BMI < 28 kg/m^2^. The adjustment was made for smoking status, BMI, sex, FPG, drinking status, AST, LDL-c, age, DBP, Scr, ALT, BUN, family history of diabetes, and SBP. The results showed that the HR between them was 0.845 (95% CI 0.816–0.875, p < 0.001). Similarly, when TG/HDL-c was treated as a categorical variable, sensitivity analyses of the multivariate-adjusted model indicated that participants in the second, third, and fourth quartiles had significantly lower rates of returning to normoglycemia from prediabetes compared to individuals in the first quartile of TG/HDL-c (Table 5).

**Table 5:** Relationship between TG/HDL-c ratio and the probability of reverting from prediabetes to normoglycemia in different sensitivity analyses

Exposure	Model I(HR,95%CI)	Model II(HR,95%CI)	Model III(HR,95%CI)
TG/HDL-c ratio	0.845 (0.816, 0.875) < 0.001	0.869 (0.841, 0.897) < 0.001	0.870 (0.839, 0.902) < 0.001
TG/HDL-c ratio quartiles			
Q1	Ref	Ref	Ref
Q2	0.916 (0.855, 0.982) 0.014	0.926 (0.865, 0.992) 0.028	0.965 (0.893, 1.043) 0.374
Q3	0.743 (0.687, 0.803) < 0.001	0.768 (0.712, 0.829) < 0.001	0.797 (0.731, 0.868) < 0.001
Q4	0.671 (0.618, 0.729) < 0.001	0.704 (0.650, 0.763) < 0.001	0.706 (0.645, 0.772) < 0.001
P for trend	< 0.001	< 0.001	< 0.001

Model I was a sensitivity analysis performed after excluding participants with BMI ≥ 28 mmol/L (N = 12,705). We adjusted age DBP, sex,, FPG, LDL-c, AST, ALT, drinking status, Scr, smoking status, BUN, SBP, and family history of diabetes

Model II was a sensitivity analysis performed on participants without a family history of diabetes (N = 14,718). We adjusted age, sex, DBP, FPG, LDL-c, AST, ALT, smoking status, Scr, smoking status, BUN, SBP, and drinking status

Model III was a sensitivity analysis performed on participants who had never consumed alcohol (N = 11,410). We adjusted age, sex, DBP, FPG, LDL-c, AST, ALT, smoking status, Scr, smoking status, BUN, SBP, and family history of diabetes

*HR* Hazard ratios, *CI* confidence, Ref: reference

Furthermore, a sensitivity analysis was performed using the E-value to evaluate the potential influence of unmeasured confounders. The computed E-value of 1.57 exceeded the relative risk of TG/HDL-c and unmeasured confounders (1.38), suggesting that the impact of unknown or unmeasured variables on the correlation between TG/HDL-c and the regression from prediabetes to normoglycemia was likely negligible. Collectively, the results of the sensitivity analyses conducted provide strong evidence of the robustness of our findings.

### Nonlinearity is addressed using a Cox proportional hazards regression model using cubic spline functions

**Fig. 5:**
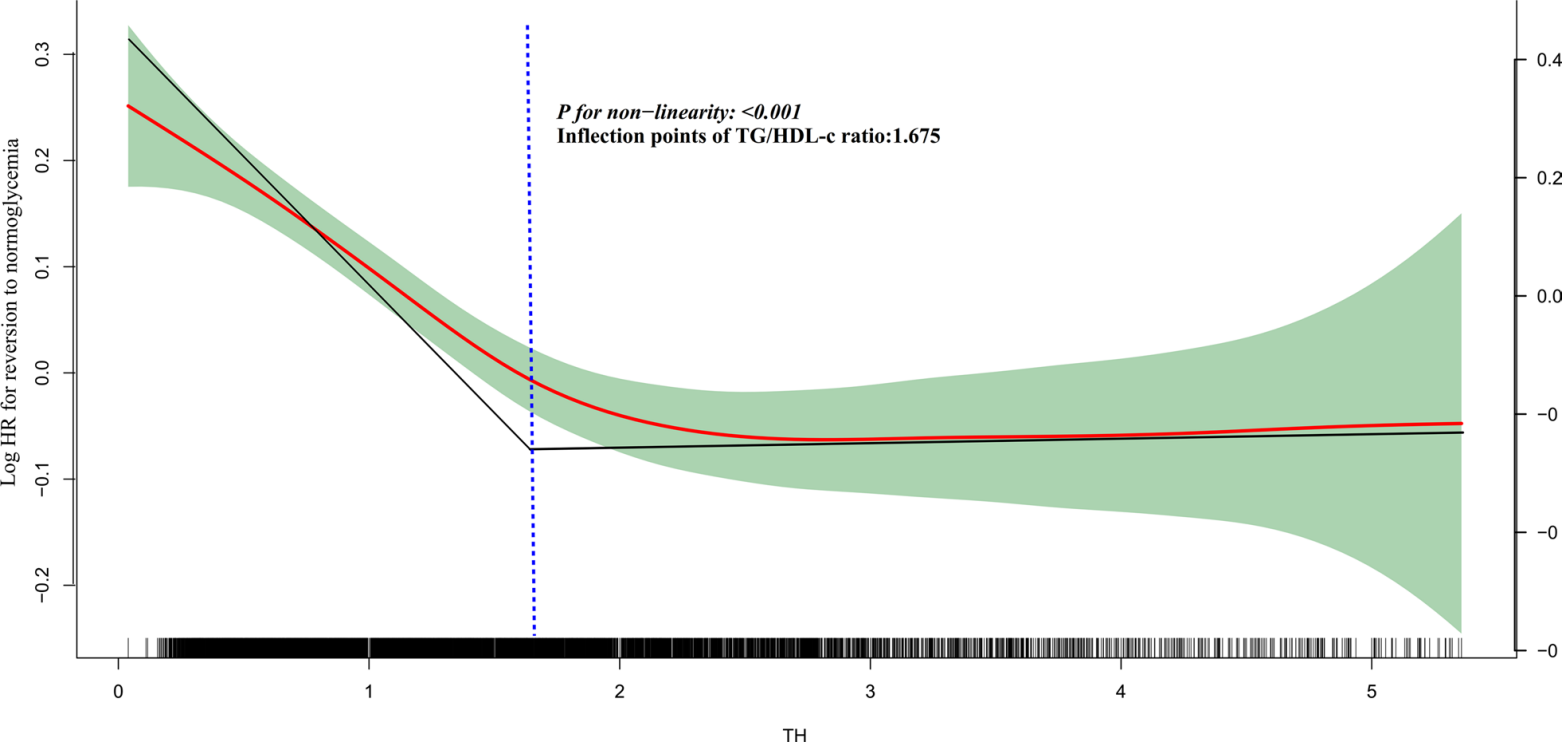
The non-linear relationship between TG/HDL-c and reversion to normoglycemia in patients with prediabetes. The relationship between TG/HDL-c and regression to normoglycemia from prediabetes was non-linear, with the inflection point of BMI being 1.675

Utilizing a Cox proportional hazards regression model with a cubic spline function, an observation was made regarding the non-linear association between the TG/HDL-c ratio and the rate of reversal to normoglycemia (Fig. 5). The log-likelihood ratio test yielded a p-value of < 0.001. Subsequently, a two-slice Cox proportional hazards regression model was used to calculate HRs and CIs on both sides of the inflection point. The HR on the left side of the inflection point was 0.748 (95% CI 0.699, 0.801), indicating a significant association. Conversely, on the right side of the inflection point, the HR was 0.969 (95% CI 0.920–1.021), but no statistical difference was observed (Table 6).

**Table 6:** The result of the two-piecewise linear regression model

Outcome: reversion to normoglycemia	HR, 95%CI	P-value
Standard Cox regression	0.869 (0.842, 0.897)	< 0.0001
Two-piecewise Cox regression		
Inflection points of TG/HDL-c ratio	1.675	
≤ 1.675	0.748 (0.699, 0.801)	< 0.0001
> 1.675	0.969 (0.920, 1.021)	0.2411
P for log-likelihood ratio test	< 0.001	

We adjusted age, SBP, BMI, sex, DBP, LDL-c, AST, FPG, ALT, Scr, family history of diabetes, BUN, smoking status, and drinking status

*HR* Hazard ratios, *CI *confidence

### Results of subgroup analysis

It was shown that characteristics such as smoking status, sex, BMI, age, SBP, and drinking status did not affect the link between TG/HDL-c and reversion to normoglycemia from prediabetes in any of the prespecified or exploratory subgroups analyzed (Additional file 1: Table S4). Specifically, no significant interaction was found between these factors and TG/HDL-c (P > 0.05 for interaction), indicating that they did not substantially impact the association between TG/HDL-c and the probability of reversal to normoglycemia.

## Discussion

This retrospective cohort study aimed to investigate the correlation between TG/HDL-c and the probability of reversal to normoglycemia in individuals with prediabetes. Our findings indicate that an elevation in TG/HDL-c is significantly linked to a reduced likelihood of returning to normoglycemia. Furthermore, a threshold effect curve was observed, revealing distinct associations between the TG/HDL-c ratio and the probability of reversal to normoglycemia on both sides of the inflection point.

The findings of one study revealed that, after one year of follow-up, 54% of individuals with prediabetes successfully reverted to normoglycemia, while 6% progressed to diabetes [14]. 22.6% of prediabetic people reverted to normoglycemia after a median follow-up of 2.5 years, according to the results of another prospective cohort trial with 491 participants [46]. Furthermore, a separate cohort study conducted in China, which included 14,231 Chinese adults, demonstrated that 44.9% of prediabetic individuals achieved normoglycemia within a 2-year timeframe [47]. During the 5-year follow-up period, 41.91% of patients with prediabetes returned to normoglycemia, according to our study. It is important to note that the rates of reversion to normoglycemia from prediabetes varied among these studies, potentially influenced by factors such as participant age, duration of follow-up, and ethnicity. Nevertheless, all studies confirmed a significant proportion of patients with prediabetes experienced successful recovery to normoglycemia. Thus, identifying the contributing factors to regression from prediabetes to normoglycemia holds particular importance in the prevention of diabetes and its associated complications.

The TG/HDL-c ratio has frequently been utilized in prior studies to evaluate insulin resistance (IR) [20, 48, 49]. The findings of numerous studies have demonstrated a positive link between the TG/HDL-c ratio and the risk of diabetes in the general population [22, 50, 51]. In a retrospective cohort study involving 114,787 participants, it was observed that a 1 unit increase in the TG/HDL-c ratio was associated with a 15.9% higher risk of developing diabetes (HR = 1.159, 95% CI 1.104–1.215) after adjusting for potential confounding factors [34]. In a cohort study including 2571 participants, Cox proportional hazards regression analysis revealed that TG/HDL-c was also an independent risk factor for diabetes after adjusting for relevant variables (HR = 1.29, 95% CI 1.14–1.47[22]. Likewise, a separate cohort study comprising 11,946 individuals without pre-existing diabetes from a rural region of China revealed that those situated in the uppermost quartile of the TG/HDL-c exhibited greater susceptibility to diabetes in comparison to those situated in the lowest quartile (HR: 2.11, 95% CI 1.55–2.86) [51]. Furthermore, a cohort study conducted in China demonstrated a noteworthy positive correlation between the TG/HDL-C ratio and the likelihood of progression from prediabetes to diabetes [21]. We, therefore, hypothesize that there may be a negative association between the TG/HDL-c ratio and the reversal of prediabetes to normoglycaemia. Our results showed that for patients with prediabetes, each 1-unit increase in the TG/HDL-c ratio was associated with a 13.1% reduction in the probability of returning to normoglycaemia (HR = 0.869,95% CI 0.842–0.897), validating our hypothesis. Meanwhile, sensitivity analyses found that this relationship persisted in participants with no family history of diabetes, a BMI < 28 kg/m^2^, and never drinking alcohol. Furthermore, competing risk multivariate Cox regression analyses found that the association between TG/HDL-c ratio and return to normoglycemia in prediabetes was consistent with the results of multivariate Cox proportional hazards regression models. The aforementioned endeavors substantiate the steadfastness of the correlation between the TG/HDL-c ratio and the regression from prediabetes to normoglycemia. The outcomes furnish a benchmark for refining lipid interventions to facilitate regression to normoglycemia and mitigate the likelihood of prediabetic patients developing diabetes.

The precise mechanisms responsible for the negative correlation between TG/HDL-c and the return to normoglycemia in patients with prediabetes remain unclear, but they could potentially be linked to insulin resistance. Extensive studies have validated the crucial role of insulin resistance in both the regression and progression of prediabetes [10, 52]. Additionally, evidence suggests an independent positive correlation between the TG/HDL-c ratio and the insulin resistance index, while demonstrating a negative correlation between the TG/HDL-c ratio and IR-adjusted β-cell function [50, 53]. Future research endeavors may shed light on the precise mechanisms involved, ultimately enhancing our understanding of the pathophysiology of prediabetes and mitigate the risk of prediabetes development.

In addition, this study documented for the first time a non-linear association and threshold effect between TG/HDL-c and the probability of returning to normoglycemia in patients diagnosed with prediabetes. The inflection point of TG/HDL-c was 1.675. For each 1-unit increase in the TG/HDL-c ratio, the probability of reversal to normoglycemia decreased by 25.2% when TG/HDL-c was below 1.675. There was no significant association between them when the ratio was greater than or equal to 1.675. The discovery of the curvilinear relationship between them is of good clinical value.

On the one hand, aggressive intervention to lower TG or raise HDL-c levels and maintain the TG/HDL-c ratio below 1.675 in patients with prediabetes may increase the probability of reversion o to normoglycemia. In addition, when the TG/HDL-c ratio was below 1.675, the probability of reversion to normoglycemia increased at a faster rate as the TG/HDL-c ratio decreased. This finding aids clinical consultations and informs decision-making processes for optimizing diabetes prevention strategies in patients with prediabetes.

This study possesses several notable strengths that warrant acknowledgment. Firstly, it represents the first investigation to explore the association between the TG/HDL-c ratio and regression to normoglycemia, specifically within a Chinese population diagnosed with prediabetes, thus offering valuable insights into this particular cohort. Secondly, the study delves into the non-linear relationship between the TG/HDL-c ratio and the probability of regression from prediabetes to normoglycemia and identifies the inflection point. Third, a robust approach of multiple imputation was employed to address the issue of missing data. This method ensures maximum statistical power while mitigating bias arising from incomplete covariate information, thus enhancing the reliability of the study's conclusions. Moreover, a comprehensive set of sensitivity analyses was conducted to further validate the findings. These analyses include the integration of continuous covariates as curves into the equation via generalized additive models (GAM), the computation of E-values to evaluate the potential influence of unmeasured confounders, and the reassessment of the relationship between TG/HDL-c and regression to normoglycemia among subgroups of participants with specific characteristics, such as those who reported no alcohol consumption, lacked a family history of diabetes and had never consumed alcohol. In addition, a multivariate Cox regression analysis of competing risks was performed, considering the development of diabetes in prediabetes as a competing risk event for return to normoglycemia.

Several potential limitations of this study should be acknowledged. Firstly, the study population consisted solely of Chinese participants, which may limit the generalizability of the findings to populations with different genetic backgrounds. Further research is warranted to investigate the association between the TG/HDL-c ratio and regression to normoglycemia in prediabetic patients from diverse ethnic backgrounds. Secondly, the definition of return to normoglycemia did not include measurements of a 2-h oral glucose tolerance test or glycated hemoglobin levels, potentially leading to an overestimation of the probability of conversion to normoglycemia in prediabetes. Future studies should consider incorporating these additional measurements to enhance the accuracy of assessing normoglycemia status. Thirdly, as this study relied on secondary analysis of published data, certain variables not included in the original dataset, such as waist circumference and insulin concentration, could not be adjusted. However, the calculated E-values were utilized to estimate the potential impact of unmeasured confounders, indicating that it is unlikely for unmeasured confounders to fully explain the observed results. In the future, we will construct our study or collaborate with other researchers to collect additional covariates. In addition, the loss-to-follow-up rate in this study seems to be high, but since this is a secondary analysis and the original data is based on a large sample retrospective cohort study, a low loss-to-follow-up rate is difficult to achieve. In the future, we could design a prospective cohort study to reduce the lost to follow-up rate even lower. Furthermore, being an observational study, this investigation established an inferred association between the TG/HDL-c ratio and return to normoglycemia rather than establishing a causal association. Caution should be exercised when interpreting the results as causal conclusions cannot be drawn solely based on this study. Finally, the study only assessed the TG/HDL-c ratio and other parameters at baseline without considering their changes over time. Future research could adopt a longitudinal design or collaborate with other researchers to obtain multiple data points, allowing for the examination of changes in the TG/HDL-c ratio over the course of patient follow-up, which would provide a more comprehensive understanding of its relationship with regression to normoglycemia in prediabetic patients.

## Conclusion

According to this study, among Chinese individuals with prediabetes, there is a positive and non-linear link between the TG/HDL-c ratio and the probability of regression to normoglycemia. Notably, a saturating effect between the TG/HDL-c ratio and the probability of reversion to normoglycemia has been identified. A robust inverse correlation is evident between the two variables when the TG/HDL-c ratio is less than 1.675. This finding is anticipated to serve as a clinical guide for managing dyslipidemia in individuals with prediabetes. Interventions aimed at reducing TG or elevating HDL-c levels are recommended to effectively lower the TG/HDL-c ratio below the inflection point. Such interventions have the potential to substantially enhance the likelihood of regression to normoglycemia in cases where the TG/HDL-c ratio is below 1.675.

### Supplementary Information

**Additional file 1: Table S1.** Collinearity screening. **Table S2**. Baseline characteristics according to reversal and progression status of patients with prediabetes. **Table S3**. The rate of reversion to normoglycemia in people with prediabetes (% or Per 1000 person-year). **Table S4.** Stratified associations between the TG/HDL-c ratio and reversion to normoglycemia in patients with prediabetes by age, BMI, sex, SBP, DBP, smoking status, and drinking status. **Figure S1.** The rate of reversion to normoglycemia in people with prediabetes stratified by quartile of TG/HDL-c. Participants with higher TG/HDL-c had a significantly lower reversal rate than those with a lower TG/HDL-c(p<0.001 for trend).

**Additional file 2.** R code for the entire statistical analysis process.

## Data Availability

The database 'DATADRYAD' (www.Datadryad.org) provides data access.

## Abbreviations

GAM: Generalized additive model
IGT: Impaired glucose tolerance
TC: Total cholesterol; Scr, serum creatinine
IFG: Impaired fasting glucose
FPG: Fasting plasma glucose
SBP: Systolic blood pressure
TG/HDL-C ratio: Triglyceride-to-high density lipoprotein cholesterol ratio
LDL-c: Low-density lipoprotein cholesterol
BUN: Blood urea nitrogen
AST: Aspartate aminotransferase
DBP: Diastolic blood pressure
BMI: Body mass index
HDL-c: High-density lipoprotein cholesterol
ALT: Alanine aminotransferase
TG: Triglyceride
HR: Hazard ratio
IDF: International Diabetes Federation
Ref: Reference
CI: Confidence interval
