# Supplementary material for: Triglyceride to high-density lipoprotein cholesterol ratio is associated with regression to normoglycemia from prediabetes in adults: a 5-year cohort study in China
Source: J Transl Med. 2023 Nov 30;21:868. doi: 10.1186/s12967-023-04752-w (PMC10688482; doi:10.1186/s12967-023-04752-w)

**Triglyceride to high-density lipoprotein cholesterol ratio is associated with regression to normoglycemia from prediabetes in adults: a 5-year cohort study in China.**

**Running title:** **TG/HDL-c and regression to normoglycemia**

Zhiqiang Huang^1#^, Yong Han^1#^, Haofei Hu^2#^, Changchun Cao^3#^ , Dehong Liu^1*^, Zhibin Wang^1*^

^1^Department of Emergency, Shenzhen Second People's Hospital, Shenzhen 518000, Guangdong Province, China

^2^Department of Nephrology, Shenzhen Second People's Hospital, Shenzhen 518000, Guangdong Province, China.

^3^Department of Rehabilitation, Shenzhen Dapeng New District Nan'ao People's Hospital, Shenzhen 518000, Guangdong Province, China

**^#^ Zhiqiang Huang, Yong Han, Haofei Hu and Changchun Cao have contributed equally to this work.**

***Corresponding author**

Dehong Liu

Department of Emergency, Shenzhen Second People's Hospital

No.3002 Sungang Road, Futian District,

Shenzhen 518035,

Guangdong Province,

China

E-mail: dhliu_emergency@163.com

***Corresponding author**

**Zhibin Wang**

Department of Emergency,

Shenzhen Second People's Hospital

No.3002 Sungang Road, Futian District,

Shenzhen 518000,

Guangdong Province,

China

E-mail: [38669029@qq.com](mailto:38669029@qq.com)

Table S1 Collinearity screening

|  | Step 1 | Step 2 | Step 3 |
| --- | --- | --- | --- |
| TG/HDL-c ratio | 15.7 | 1.8 | 1.5 |
| DBP (mmHg) | 1.8 | 1.8 | 1.8 |
| BMI (kg/m^2^) | 1.3 | 1.3 | 1.3 |
| TC (mmol/L) | 6.8 | 6.8 | NA |
| SBP (mmHg) | 1.7 | 1.7 | 1.7 |
| AST(U/L) | 3.1 | 3.1 | 3.1 |
| BUN (mmol/L) | 1.1 | 1.1 | 1.1 |
| LDL-c(mmol/L) | 5.8 | 5.8 | 1.1 |
| Scr (μmol/L) | 1.2 | 1.2 | 1.2 |
| Smoking status | 1.1 | 1.1 | 1.1 |
| ALT(U/L) | 3.2 | 3.2 | 3.2 |
| Drinking status | 1.1 | 1.1 | 1.1 |
| Family history of diabetes | 1 | 1 | 1 |

Variables excluded from collinearity screening: TC

Abbreviations: DBP, diastolic blood pressure; BMI, body mass index; TC, total cholesterol, SBP, systolic blood pressure; TG triglyceride, BMI, body mass index; AST aspartate aminotransferase; LDL-c, low-density lipid cholesterol; ALT, alanine aminotransferase; BUN, blood urea nitrogen; HDL-c, high-density lipoprotein cholesterol; Scr, serum creatinine.

**Table S2 Baseline characteristics according to reversal and progression status of patients with prediabetes**

|  | Persistent prediabetes | reversion to normoglycemia | Progression to diabetes | P-value |
| --- | --- | --- | --- | --- |
| N | 7023 | 6332 | 1752 |  |
| Age(years) | 52.576 ± 13.005 | 47.476 ± 13.400 | 56.946 ± 12.258 | <0.001 |
| Height(cm) | 166.551 ± 8.330 | 166.654 ± 8.411 | 166.615 ± 8.432 | 0.776 |
| Weight(kg) | 69.733 ± 11.828 | 67.478 ± 12.100 | 72.620 ± 12.550 | <0.001 |
| BMI (kg/m^2^) | 25.042 ± 3.216 | 24.187 ± 3.284 | 26.048 ± 3.342 | <0.001 |
| SBP (mmHg) | 129.199 ± 17.722 | 124.101 ± 16.903 | 132.405 ± 18.201 | <0.001 |
| FPG(mmol/L) | 5.980 ± 0.311 | 5.834 ± 0.238 | 6.262 ± 0.370 | <0.001 |
| DBP (mmHg) | 79.475 ± 11.212 | 76.707 ± 10.844 | 80.511 ± 11.367 | <0.001 |
| TC (mmol/L) | 5.058 ± 0.932 | 4.975 ± 0.931 | 5.105 ± 0.962 | <0.001 |
| TG (mmol/L) | 1.724 ± 1.011 | 1.540 ± 0.946 | 1.993 ± 1.152 | <0.001 |
| LDL-c(mmol/L) | 2.962 ± 0.709 | 2.921 ± 0.715 | 2.955 ± 0.710 | 0.003 |
| HDL-c(mmol/L) | 1.332 ± 0.287 | 1.362 ± 0.295 | 1.304 ± 0.358 | <0.001 |
| ALT(U/L) | 28.235 ± 24.394 | 25.796 ± 19.531 | 34.123 ± 27.303 | <0.001 |
| AST(U/L) | 26.488 ± 12.431 | 24.971 ± 10.114 | 29.133 ± 13.513 | <0.001 |
| BUN (mmol/L) | 5.051 ± 1.225 | 4.938 ± 1.248 | 5.051 ± 1.264 | <0.001 |
| Scr (μmol/L) | 73.746 ± 15.961 | 72.017 ± 16.265 | 73.201 ± 16.492 | <0.001 |
| Sex |  |  |  | <0.001 |
| Male | 4662 (66.382%) | 3826 (60.423%) | 1257 (71.747%) |  |
| Female | 2361 (33.618%) | 2506 (39.577%) | 495 (28.253%) |  |
| Smoking status |  |  |  | 0.504 |
| Current smoker | 1763 (25.103%) | 1587 (25.063%) | 431 (24.600%) |  |
| Ever smoker | 366 (5.211%) | 301 (4.754%) | 76 (4.338%) |  |
| Never | 4894 (69.685%) | 4444 (70.183%) | 1245 (71.062%) |  |
| Drinking status |  |  |  | 0.080 |
| Current drinker | 353 (5.026%) | 291 (4.596%) | 109 (6.221%) |  |
| Ever drinker | 1386 (19.735%) | 1221 (19.283%) | 337 (19.235%) |  |
| Never | 5284 (75.239%) | 4820 (76.121%) | 1306 (74.543%) |  |
| Family history of diabetes |  |  |  | 0.148 |
| No | 6834 (97.309%) | 6165 (97.363%) | 1719 (98.116%) |  |
| Yes | 189 (2.691%) | 167 (2.637%) | 33 (1.884%) |  |

Abbreviations: DBP, diastolic blood pressure; BMI, body mass index; TC, total cholesterol, SBP, systolic blood pressure; TG triglyceride, BMI, body mass index; AST aspartate aminotransferase; LDL-c, low-density lipid cholesterol; ALT, alanine aminotransferase; BUN, blood urea nitrogen; HDL-c, high-density lipoprotein cholesterol; Scr, serum creatinine.

**Table S3** **The rate of reversion to normoglycemia in people with prediabetes (% or** Per 1000 person-year**)**

| TG/HDL-c ratio | Participants(n) | Reversion events(n) | | Reversal rate (95% CI) (%) | Per 1000 person-year |
| --- | --- | --- | --- | --- | --- |
| Total | 15107 | | 6332 | 41.91(41.13-42.70) | 142.21 |
| Q1(<0.692) | 3764 | | 1940 | 51.54 (49.94-53.14) | 179.85 |
| Q2(0.692-1.093) | 3789 | | 1707 | 45.05(43.47-46.64) | 155.35 |
| Q3(1.092-1.719) | 3777 | | 1372 | 36.33 (34.79-37.86) | 122.95 |
| Q4(≥1.719) | 3777 | | 1313 | 34.76(33.24-36.28) | 113.15 |
| P for trend |  | |  | <0.001 |  |

TG/HDL-C ratio, triglyceride-to-high density lipoprotein cholesterol ratio; CI: confidence interval,

**Table S4. Stratified associations between the TG/HDL-c ratio and reversion to normoglycemia in patients with prediabetes by age, BMI, sex, SBP, DBP, smoking status, and drinking status.**

| Characteristic | No of participants | HR (95%CI) P value P for interaction |
| --- | --- | --- |
| Age, years  <30  30 to <40  40 to <50  50 to <60  60 to <70 | 589  3047  3387  3818  2916 | 0.1714  0.911 (0.786, 1.055) 0.2122  0.937 (0.882, 0.995) 0.0329  0.840 (0.790, 0.892) <0.0001  0.817 (0.767, 0.870) <0.0001  0.894 (0.831, 0.961) 0.0024 |
| ≥70 | 1350 | 0.902 (0.794, 1.025) 0.1129 |
| BMI(kg/m^2^) |  | 0.4786 |
| <18.5 | 257 | 0.916 (0.630, 1.332) 0.6460 |
| 18.5-24 | 5948 | 0.844 (0.800, 0.889) <0.0001 |
| 24-28 | 6500 | 0.870 (0.831, 0.911) <0.0001 |
| >=28 | 2402 | 0.905 (0.841, 0.975) 0.0082 |
| Sex |  | 0.2535 |
| Male | 9745 | 0.878 (0.847, 0.911) <0.0001 |
| Female | 5362 | 0.845 (0.797, 0.896) <0.0001 |
| SBP (mmHg) |  | 0.2478 |
| <140 | 11736 | 0.861 (0.831, 0.892) <0.0001 |
| ≥140 | 3371 | 0.901 (0.841, 0.964) 0.0026 |
| Smoking status |  | 0.1692 |
| Current smoker | 3781 | 0.909 (0.859, 0.962) 0.0010 |
| Ever smoker | 743 | 0.845 (0.736, 0.971) 0.0174 |
| Never | 10583 | 0.855 (0.823, 0.888) <0.0001 |
| Drinking status |  | 0.5778 |
| Current drinker | 753 | 0.838 (0.730, 0.961) 0.0113 |
| Ever drinker | 2944 | 0.845 (0.786, 0.909) <0.0001 |
| Never | 11410 | 0.876 (0.846, 0.907) <0.0001 |

Note 1: Above model adjusted for age, sex, BMI, SBP, DBP, FPG, LDL-c, ALT, AST, Scr, BUN, smoking status, drinking status, family history of diabetes.

Note 2: In each case, the model is not adjusted for the stratification variable.

HR, Hazard ratios; CI: confidence, Ref: reference.

**Figure S1. The rate of reversion to normoglycemia in people with prediabetes stratified by quartile of TG/HDL-c.** Participants with higher TG/HDL-c had a significantly lower reversal rate than those with a lower TG/HDL-c(p<0.001 for trend).


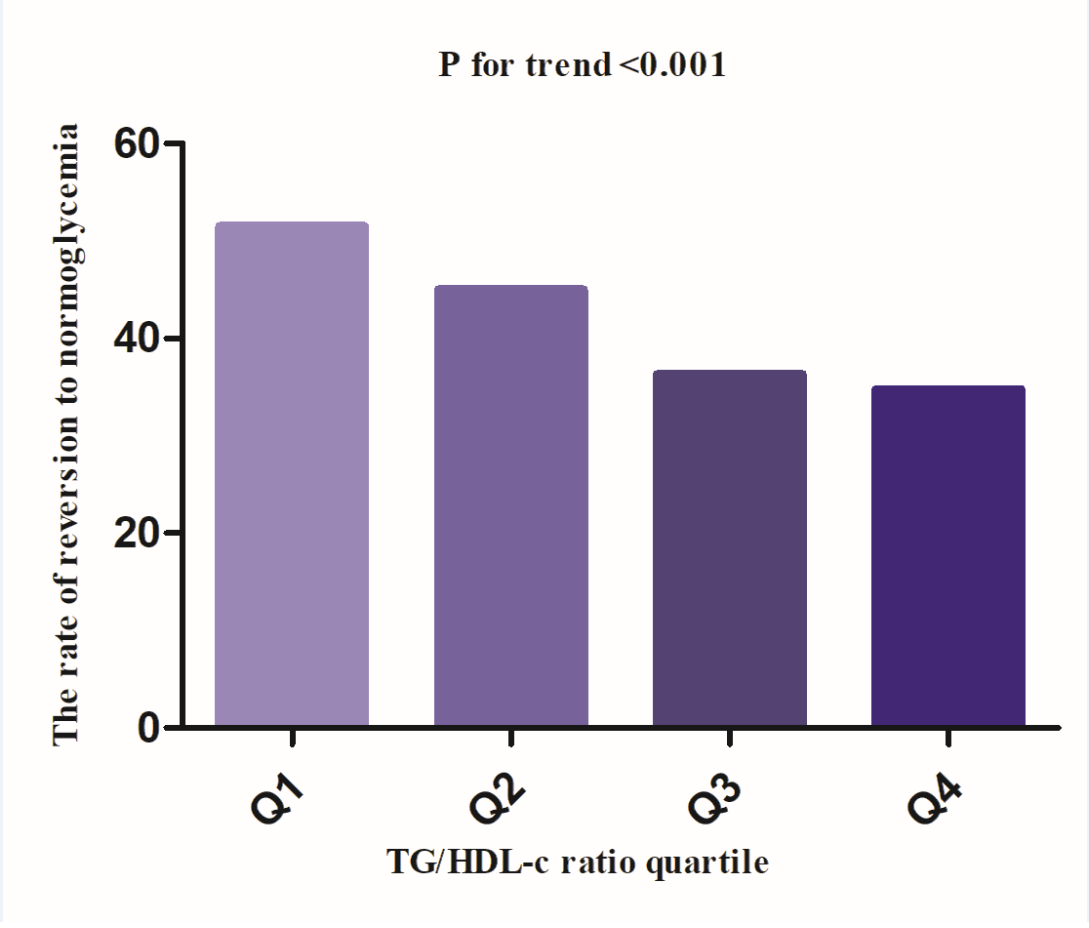

Supplement: Supplementary file 1 — Additional file 1: Table S1. Collinearity screening. Table S2. Baseline characteristics according to reversal and progression status of patients with prediabetes. Table S3. The rate of reversion to normoglycemia in people with prediabetes (% or Per 1000 person-year). Table S4. Stratified associations between the TG/HDL-c ratio and reversion to normoglycemia in patients with prediabetes by age, BMI, sex, SBP, DBP, smoking status, and drinking status. Figure S1. The rate of reversion to normoglycemia in people with prediabetes stratified by quartile of TG/HDL-c. Participants with higher TG/HDL-c had a significantly lower reversal rate than those with a lower TG/HDL-c(p<0.001 for trend). [file 12967_2023_4752_MOESM1_ESM.docx]
